# Flexible PBAT-Based Composite Filaments for Tunable FDM 3D Printing

**DOI:** 10.1021/acsabm.2c00203

**Published:** 2022-06-22

**Authors:** Corrado Sciancalepore, Elena Togliatti, Marina Marozzi, Federica Maria Angela Rizzi, Diego Pugliese, Antonella Cavazza, Olimpia Pitirollo, Maria Grimaldi, Daniel Milanese

**Affiliations:** † Dipartimento di Ingegneria e Architettura, Università di Parma, Parco Area delle Scienze 181/A, 43124 Parma, Italia; ‡ Dipartimento di Medicina e Chirurgia, Università di Parma, Via Volturno 39/E, 43126 Parma, Italia; § Dipartimento di Scienza Applicata e Tecnologia, Politecnico di Torino, Corso Duca degli Abruzzi 24, 10129 Torino, Italia; ∥ INSTM, Consorzio Interuniversitario Nazionale per la Scienza e la Tecnologia dei Materiali, Via G. Giusti 9, 50121 Firenze, Italia; ⊥ Dipartimento di Scienze Chimiche, della Vita e della Sostenibilità Ambientale, Università di Parma, Parco Area delle Scienze 17/A, 43124 Parma, Italia

**Keywords:** biocomposite, poly(butylene adipate-*co*-terephthalate), zein-titanium dioxide complex, 3D printing, fused deposition modeling

## Abstract

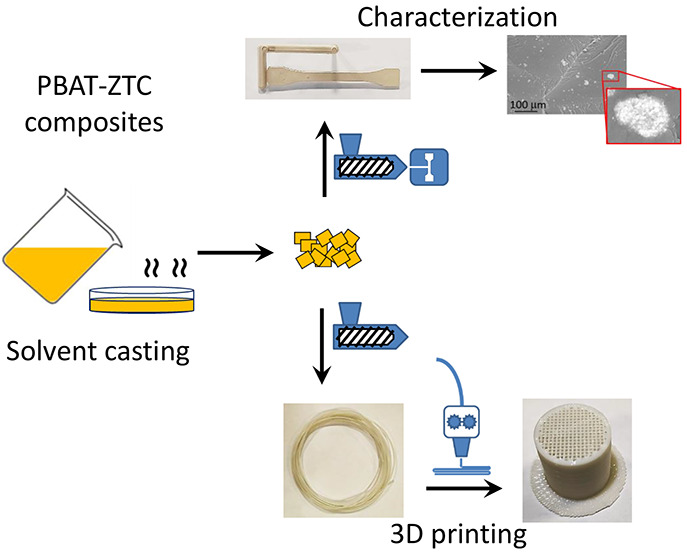

Biobased composites with peculiar properties offer an attractive route for producing environmentally friendly materials. The reinforcement for poly(butylene adipate-*co*-terephthalate) (PBAT), based on zein-titanium dioxide (TiO_2_) complex (ZTC) microparticles, is presented and used to produce composite filaments, successfully 3-dimensionally (3D) printed by fused deposition modeling (FDM). The outcome of ZTC addition, ranging from 5 to 40 wt %, on the thermo-mechanical properties of composite materials was analyzed. Results reveal that storage modulus increased with increasing the ZTC content, leading to a slight increase in the glass transition temperature. The creep compliance varies with the ZTC concentration, denoting a better resistance to deformation under constant stress conditions for composites with higher complex content. Scanning electron microscopy was used to assess the quality of interphase adhesion between PBAT and ZTC, showing good dispersion and distribution of complex microparticles in the polymer matrix. Infrared spectroscopy confirmed the formation of a valid interface due to the formation of hydrogen bonds between filler and polymer matrix. Preliminary tests on the biocompatibility of these materials were also performed, showing no cytotoxic effects on cell viability. Finally, the 3D printability of biobased composites was demonstrated by realizing complex structures with a commercial FDM printer.

## 1 Introduction

The transition from a linear to a circular economy can be accelerated by promoting the use of bioplastics, materials that are biobased, biodegradable, or both.^1^ Bioplastics currently represent only a small fraction of all the plastic-based materials annually produced in the world. However, the bioplastic market is constantly expanding, and biobased alternatives to traditional plastics are available in many fields, from agri-food to biomedical applications.^1,2^

Poly(butylene adipate-*co*-terephthalate) (PBAT) is a biodegradable thermoplastic polyester derived mainly from fossil sources but with good potential for more environmentally sustainable production.^3−5^ It consists of two different repeating units, butylene terephthalate (BT) and butylene adipate (BA), joined together by a condensation reaction, and their molar ratio influences the properties of the copolymer.^6^

The perspective given by bioplastics can be expanded by the production of biocomposites, thus obtaining a whole series of sustainable materials with improved and diversified properties adaptable to a wider range of applications.

There are high expectations on eco-friendly reinforced plastics because they merge sustainability and competitive properties into a single material.

Biobased advanced composite materials can be produced from biopolymers reinforced with suitable fillers or nanofillers.^7^ In this way, the improvement in the structural and functional properties of the biopolymers is combined with a reduction of the carbon footprint of traditional plastics. The fillers include natural fibers, metals, and metal oxides based on their application. Among all natural-derived reinforcing agents, polysaccharides are the most representative group, which also include cellulose, lignin, hemicellulose, chitosan, starch, and alginate.^8,9^ Frequently used inorganic reinforcements are silver nanoparticles, carbon nanotubes, micro- or nanostructured alumina (Al_2_O_3_), silica (SiO_2_), and titania (TiO_2_) for a broad range of applications, from biomedical to packaging one.^10^

TiO_2_ has been used as a strengthening agent to develop organic–inorganic hybrid materials with improved physicochemical,^11^ mechanical, ultraviolet (UV),^12^ gas barrier, water-resistance, and antimicrobial properties.^13,14^ The use of TiO_2_ as a reinforcing agent for biopolymer matrices is particularly interesting because of its nontoxicity, biocompatibility, and sustainability.^15,16^

However, the polymeric matrix and the inorganic filler may not be completely compatible: poor interactions at the interface would reduce the mechanical properties of the final composite material. Modification or functionalization of reinforcing fillers is the key factor to implement the interface interactions between reinforcing agents and polymer matrix and successfully obtain high-performance composite materials, comparable with conventional oil-based polymer composite counterparts. To ensure good adhesion between filler and matrix and an effective stress transfer, it is possible to use a compatibilization agent, usually consisting of synthetic molecules, such as maleic anhydride, structures based on epoxy, and isocyanate groups or radical compounds, capable of bridging the chemical functionalities of filler and polymer matrix.^17,18^

Proteins are natural amphiphilic compounds containing both hydrophilic and lipophilic segments, that can be used respectively as a biocoupler with hydrophilic groups, closer to the inorganic and polar systems, and hydrophobic functionalities more suitable for polymer characteristics. In fact, the protein lipophilic segments are shielded by hydrophilic parts in polar environments, but in the presence of apolar and oleophilic characteristics, they partially unfold and adsorb strongly at the organic/inorganic interface.^19^

Zein is a protein deriving from the industrial scraps of corn processing.^20^ Being a renewable natural biopolymer, pure zein is difficult to process because it does not show a thermoplastic behavior and exhibits extremely poor mechanical properties.^21,22^

In this work, zein is employed as a coupling agent and cofiller to prepare a complex structure with TiO_2_. Thus, the zein-TiO_2_ complex (ZTC) represents a hybrid organic–inorganic filler,^13^ able to enhance the interfacial adhesion and interaction between the inorganic reinforcement and polymer matrix. ZTC is added into PBAT by a solvent casting approach to develop eco-friendly biocomposite materials with improved and tunable mechanical properties. The influence of ZTC on the morphological, structural, thermo-mechanical, and viscoelastic properties of the composites is investigated as a function of ZTC content.

The developed biocomposite materials were also applied to the production of filaments, with tailored properties and suitability for 3D printing.

Fused deposition modeling (FDM) is an extrusion-based additive manufacturing technique enabling the creation of solid objects from digital 3D models, depositing layer-by-layer a thermoplastic filament.^23^ Commonly used polymer materials are poly(lactic acid) (PLA) or other fossil-based materials and nondegradable polymers such as poly(acrylonitrile-butadiene-styrene) (ABS).^24^

Such materials are significantly rigid, and only a few materials are available today with characteristics of elasticity and flexibility, such as synthetic polyurethane-based filaments and thermoplastic elastomers.^25^

The developed composites, based on PBAT, allow combining high structural flexibility with the production of environmentally friendly filaments for 3D printing, launching interesting challenges in the manufacturing of 3D objects with customized characteristics, shape, and dimensions.^26−28^ Indeed the biocomposite materials made by 3D printing can result in the improved and tailored performance of 3D-printed components and objects, thus reducing the gap among design, manufacturing of a particular device, and a more sustainable technological approach. In fact, 3D printing technology with biomaterials can potentially create a fully sustainable and circular manufacturing process, realizing personalized products locally only when needed and using biomaterials as feedstock.

To expand the spectrum of materials available for different applications, the use of unconventional polymers for the development of filaments with peculiar properties in FDM printing is still an open challenge. To the knowledge of the authors, little research has been carried out on the FDM 3D printability of PBAT biocomposites, mainly concerning composites with low filler content and mechanical properties poorer than those of pristine PBAT, presumably due to the limited compatibility between the filler and the matrix.^29^

This study reports on the development of biobased composite filaments at high ZTC content (5 to 40 wt %), where zein was used to raise the interaction between the filler and the matrix and improve the structural properties of the final composite. These flexible filaments were then 3D printed to produce complex and completely biobased solid systems, with remarkable biocompatibility properties, according to the carried-out cytotoxicity tests. The advantages of these eco-friendly materials can thus be combined with the production of customizable design objects by additive manufacturing, with numerous potential applications in biomedical and healthcare research.

## 2 Materials and Methods

### 2.1 Sample Preparation

Poly(butylene adipate-*co*-terephthalate) (PBAT – PBAT Ecoworld), as white granules, was bought from MAgMa Spa (Italy). Zein (CAS no. 9010-66-6) and TiO_2_ (CAS no. 13463-67-7) were acquired respectively from Sigma-Aldrich (Merck KGaA, Germany) and Carlo Erba (Johnson & Johnson, USA).

ZTC was obtained as follows: zein (50 g) was dissolved in ethanol (200 mL, Sigma-Aldrich) under magnetic stirring at the temperature of 50 °C, and TiO_2_ powder (50 g) was then gradually added. Reagents were used as-received without further modifications. When the solution became white and homogeneous, the mixture was cast and dried in an oven at 60 °C for 16 h to obtain a thin film. The ZTC film was reduced to a powder with dimensions below 25 μm by grinding (Pulverisette 0 ball mill, Fritsch, Germany) and subsequent sieving.

The PBAT-ZTC composites were solvent cast, using chloroform (CHCl_3_, Sigma-Aldrich) as a suitable solvent. PBAT grains were dissolved in CHCl_3_, and the filler microparticles were dispersed in the polymer solution. After CHCl_3_ evaporation, the solid composite films were reduced to pellets, which were heat-treated in an oven at about 60 °C until constant weight was reached. The ZTC content in the composites varied from 0 to 40 wt %, and the obtained formulations, containing respectively 0 (pure PBAT), 5, 10, 20, and 40 wt % of ZTC, were labeled as PBAT, PBAT+ZTC 5%, PBAT+ZTC 10%, PBAT+ZTC 20%, and PBAT+ZTC 40%.

The PBAT-ZTC pellets were injection molded in dumbbell specimens with a MegaTech H7/18-1 machine (Tecnica Duebi, Italy). The obtained 1BA models, as required by the UNI EN ISO 527 standard, are used for thermal, mechanical, and structural characterization.

The molding parameters are presented in Table 1.

**Table 1:** Injection Molding Process Settings

injection molding conditions	
Hopper temperature	80 °C
screw temperature	130 °C
barrel temperature	130 °C
die temperature	130 °C
injection pressure	120 bar
holding pressure	60 bar
holding time	5 s
cooling time	10 s

The PBAT-ZTC pellets were also employed to produce composite filaments for 3D printing through a single screw extrusion system (Felfil Evo, Felfil, Italy), equipped with a cooling fan array, to cool down the polymer melt, and a spooler, equipped with an optical sensor, to collect the produced filament with constant diameter. The extruder temperature was set at 150 ± 10 °C, and the screw speed was kept at 3 rpm. The filament diameter was fixed at 1.65 ± 0.10 mm with a collection speed of approximately 0.8 ± 0.1 m/min.

### 2.2 Dynamic-Mechanical Analysis

Rectangular specimens (dimensions 5 × 2 × 30 mm^3^), obtained from injection-molded models, were inserted into a single cantilever clamp and characterized by dynamic-mechanical analysis (DMA - TA 800Q DMA, TA Instruments, USA).

Dynamic storage (*E*′) and loss (*E*′′) moduli were measured from −55 to 60 °C with a temperature ramp of 3 °C/min, setting a sinusoidal strain of 10 μm and a frequency of 1 Hz. The damping factor (tan δ) was obtained by the ratio between *E*′′ and *E*′, and its peak value has been identified as the glass transition temperature (*T*_*g*_).

The creep curves of pristine and filled PBAT-based composites were also acquired in the creep experiments. The compliance, *J*(*t*) [μm^2^/N], was obtained from the ratio between the experimental strain and the constant applied stress (0.2 MPa) and represented as a function of the test time (10 min) at a fixed temperature (isothermal steps of 10 °C in the thermal range −10/60 °C). The composite material behavior for long stress times was predicted employing the time–temperature superimposition (TTS) principle: the master curves at 20 °C, as reference temperature, were generated from the creep data.^30^

### 2.3 Particle Size Analysis

The laser granulometry (Mastersizer 3000 laser granulometer, Malvern Instruments Ltd., UK) was used to obtain information on the ZTC particle size distribution. The particles were dispersed in water, and the angular scattering intensity data were interpreted by the Fraunhofer’s theory. The ZTC volume distribution was represented by the *D10*, *D50*, and *D90* standard percentiles, under which 10, 50, and 90% of the sample size respectively fall. The equivalent volume mean diameter, *D*_*mean*_, is the parameter used to indicate the average size of ZTC particles.

### 2.4 Infrared Spectroscopy

The composite structural variations were identified by infrared (IR) spectroscopy in attenuated total reflectance (ATR) mode. Specimen spectra were collected between 4000 and 400 cm^–1^ with the Spectrum Two FT-IR spectrophotometer (PerkinElmer, USA). The average of 16 scans and the resolution of 2 cm^–1^ were set to obtain well-defined spectra.

### 2.5 Scanning Electron Microscopy and Electron Dispersion Spectroscopy

Scanning electron microscopy (SEM) images were recorded to investigate the PBAT-ZTC composite microstructures and ZTC powder morphology. The analyzed surfaces were obtained by breaking under cryogenic conditions and coated with a nanometric gold layer to make them conductive.

The Nova NanoSEM 450 microscopy (FEI company, USA) was employed in backscattered electron mode to highlight the compositional contrast of the samples. 15 [kV] as acceleration voltage, 4 [a.u.] as spot size, and 6 [mm] as working distance are the main settings used during all measurements.

Elemental composition analysis was performed using a QUANTAX-200 energy-dispersive X-ray spectroscopy system (EDS) (Bruker, Germany), inserted in the SEM instrumental configuration, allowing the recording of the sample elemental composition.

### 2.6 Fused Deposition Modeling 3D Printing

The extruded filaments of pure and loaded PBAT were used for the FDM 3D printing of two types of objects: a scaffold as an example of complex geometry and a ring to macroscopically highlight the possible variations and customization of the mechanical behavior due to the different composite material composition. The printing head temperature was set in the range of 150–170 °C, while the printing speed was 20 mm/s. A nozzle with an extrusion diameter of 0.6 mm was used to print a layer height of 0.2 mm.

Scaffolds had a cylindrical geometry with diameter and height of 20 mm, printed with a fill density of 45% and a grid infill pattern with adjacent layers offset by an angle of 90°. The scaffold wall was set with a width of three concentric shells, overlapped by 50%. A 6-loop brim was selected as a platform to ensure better adhesion of the object to the printing base.

Rings have a diameter of 25 mm and a height of 3 mm. The ring thickness was reached by depositing three concentric shells, overlapped by 50%. Also in this case, a brim type support was used. In addition to pure PBAT and various PBAT-based composites, a poly(lactic acid) (PLA - Raised3D Premium filament) ring was also printed for the sake of comparison.

### 2.7 Biocompatibility Test

Human dermal fibroblasts (HDFs) were seeded in a 6-well plate (5 × 10^4^ cells/mL) in RPMI-1640 medium (Gibco, Life Technologies, Canada) supplemented with 10% fetal bovine serum (FBS), 2 mM l-glutamine, 100 U/mL penicillin, and 100 g/mL streptomycin (Gibco, Life Technologies, Canada) and maintained at 37 °C in a humidified atmosphere supplied with 5% CO_2_. Once attached, the cells were treated with sterile particles (2.5 mg/mL) of ZTC, PBAT, and PBAT-based composites with different ZTC content (PBAT+ZTC 5%, PBAT+ZTC 10%, PBAT+ZTC 20%, PBAT+ZTC 40%) for 24, 48, and 96 h. Cell images were acquired via the transmitted light microscope (TLM) Axiovert 200 (Carl Zeiss, Gottingen, Germany) at 10× magnification.

Cell viability was evaluated by the CellTiter-Glo luminescent cell viability assay (Promega, USA) based on the quantitation of adenosine triphosphate (ATP for detection of viable and metabolically active cells). Briefly, cells were seeded in a 96-well plate (5 × 10^4^ cells/mL) and allowed to attach. The day after, the cells were treated with ZTC, PBAT, and PBAT-based composites, as previously specified. The total ATP intracellular content of viable cells was determined after 24 and 48 h following the CellTiter-Glo luminescent cell viability assay manufacturer’s protocol. The luminescent signal was measured by the EnSpire multimode plate reader instrument (PerkinElmer, USA). The cell viability of treated cells was expressed as % value of the viability measured in the CellTiter-Glo luminescent cell viability assay of untreated cells (CTRL). All measurement data were statistically examined with the one-way analysis of variance (ANOVA) to compare experimental variability values between multiple groups. Differences with the control were considered statistically significant at a probability P-value <0.01 (significant level α = 0.01, two-sided confidence interval).

## 3 Results and Discussion

### 3.1 DMA Characterization

**Figure 1:**
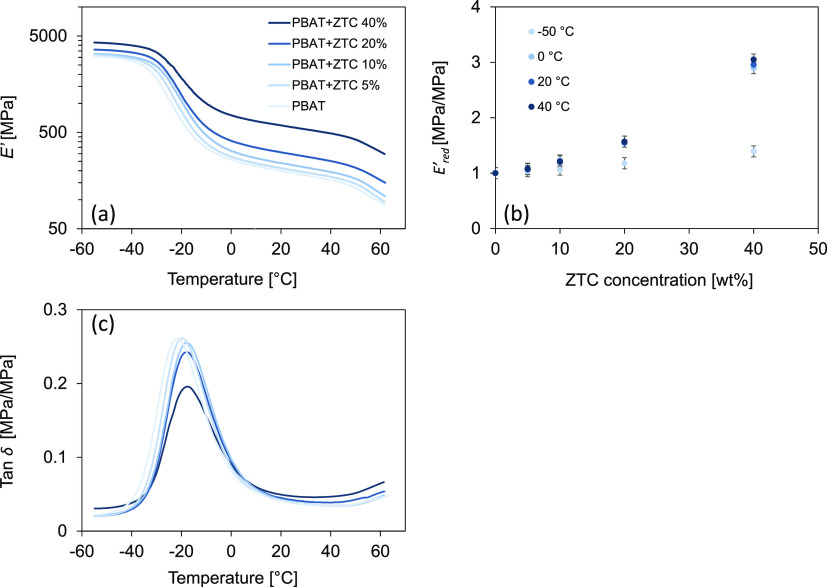
Storage modulus (*E*′) as a function of temperature for composite materials at different ZTC content (a); reduced storage modulus (*E*′_*red*_) as a function of ZTC concentration at different fixed temperatures (b); and tan δ as a function of temperature for composites with different ZTC content (c).

Figure 1(a) shows the trend of *E*′ [MPa] as a function of temperature for the composite materials with increasing ZTC content.

At low temperatures, all specimens show an increase in *E*′, between 3.0 ± 0.2 and 4.3 ± 0.2 [GPa] going from pristine PBAT to PBAT+ZTC 40%. In each curve, the *E*′ values exhibit an accentuated inflection near a temperature of about −20 °C, a value corresponding to the glass transition, where polymer chains experience an initial degree of movement. In the rubbery state, *E*′ decreases with increasing the temperature for all samples. However, the strengthening trend is preserved, with *E*′ ranging from 200 ± 25 to 590 ± 40 [MPa] at 20 °C for unloaded PBAT and PBAT+ZTC 40%, respectively. Above 50 °C, the additional bending of *E*′ can be ascribed to the initial melting of the crystalline fraction in the polymer matrix, leading to a further decrease in viscosity. Beyond this limit, the material can no longer be considered a solid.

Overall, an increase in *E*′, with increasing the filler content in the composite, is observed in the whole analyzed thermal range. However, the *E*′ increment is more evident at temperatures above *T*_*g*_, as highlighted in Figure 1(b), showing the reduced modulus, *E*′_*red*_, as a function of the ZTC content at different temperatures. *E*′_*red*_ is the ratio between the storage moduli of the composite and the pure PBAT, respectively, at a fixed temperature. By reporting *E*′_*red*_ as a function of the filler content, the obtained trend indicates the greater incidence of the filler at temperatures higher than the glass transition. In addition, the reinforcement strength remains almost constant between 0 and 40 °C, effectively increasing the thermal range for the composite materials application.

The reinforcing effect that results in the increased structural stiffness may be associated with the reduced translational freedom of the polymer chains, due to both the physical hindrance of the rigid ZTC particles and the higher density of chemical interactions between the polymer matrix and the protein structures.^31^

Tan δ trend for composites with different filler content, as a function of temperature, is shown in Figure 1(c). A progressive decrease in peak height, associated with a slight shift toward higher temperatures, can be observed with increasing filler content. This behavior can be related to the increase in effective filler–matrix interactions, which are intensified as filler content increases, and consequently to the decrease of free polymer chains fraction.^31^

**Figure 2:**
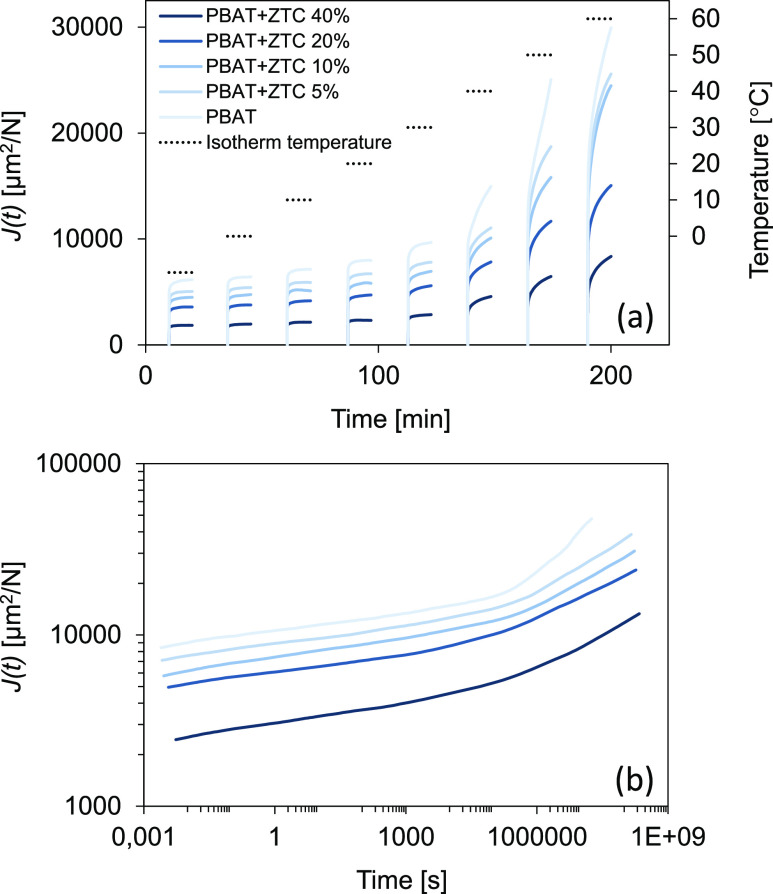
Creep compliance (*J*(*t*)) curves for PBAT-based composites at different isothermal temperatures (a) and master curves of PBAT-based composites generated by the TTS principle at 20 °C (b).

*J*(*t*) curves at different temperatures (from −10 to 60 °C, with thermal steps of 10 °C) for PBAT-based composites are reported in Figure 2(a), where it is evident how the ZTC complex can significantly increase the creep resistance. In fact, *J*(*t*) decreases from 8.1 ± 0.3 to 2.3 ± 0.1 [GPa^–1^] at 20 °C, going from the pristine PBAT to PBAT+ZTC 40%, respectively. The creep resistance difference between neat PBAT and composites becomes greater with increasing ZTC content and temperature.

At temperatures above 20 °C, the increase in the slope of the viscous section and the increase in *J*(*t*) values are particularly evident in the creep curves, especially for composites with a low ZTC concentration. In fact, for these composites, the lack of interchain bonds, determined by the filler, is such that they do not hinder the flow of polymer chains. From a practical point of view, this mechanical enhancement is particularly useful for composites involved in long-time operations.^32^

Generally, the isothermal *J*(*t*) curves can be described by the Burgers’ model, composed of a linear combination of Maxwell’s and Kelvin’s elements.^33^ The creep compliance formula of this model is given by

, where *E*_*M*_ and *η*_*M*_ are the elastic and viscous parameters of Maxwell’s unit; meanwhile, *E*_*K*_ and *η*_*K*_ are the elastic and viscous parameters of Kelvin’s unit.

Based on Burgers’ model, the change in creep curves and the general *J*(*t*) reduction, due to the ZTC addition, result in an increase of Maxwell’s parameters (*E*_*M*_ and *η*_*M*_), leading to the reduction in elastic strain and viscous flow and an increase of the exponential parameter in Kelvin’s dashpot (), determining the reduction of the retarded elastic strain.

The strengthened creep resistance mechanism (lower strain and slower deformations) was also confirmed by the master curves generated at 20 °C using the Williams–Landel–Ferry’s model, as shown in Figure 2(b).^30^

Through the TTS principle, the *J*(*t*) behavior at 20 °C of all prepared PBAT-based composite materials can be estimated and predicted for a much longer time than laboratory test duration (up to 10 years). The final *J*(*t*) values are again much lower in filled polymers for the whole-time range, giving the possibility to modulate and extend the use of these materials for more time and in more fields.

### 3.2 SEM and EDS Characterization

**Figure 3:**
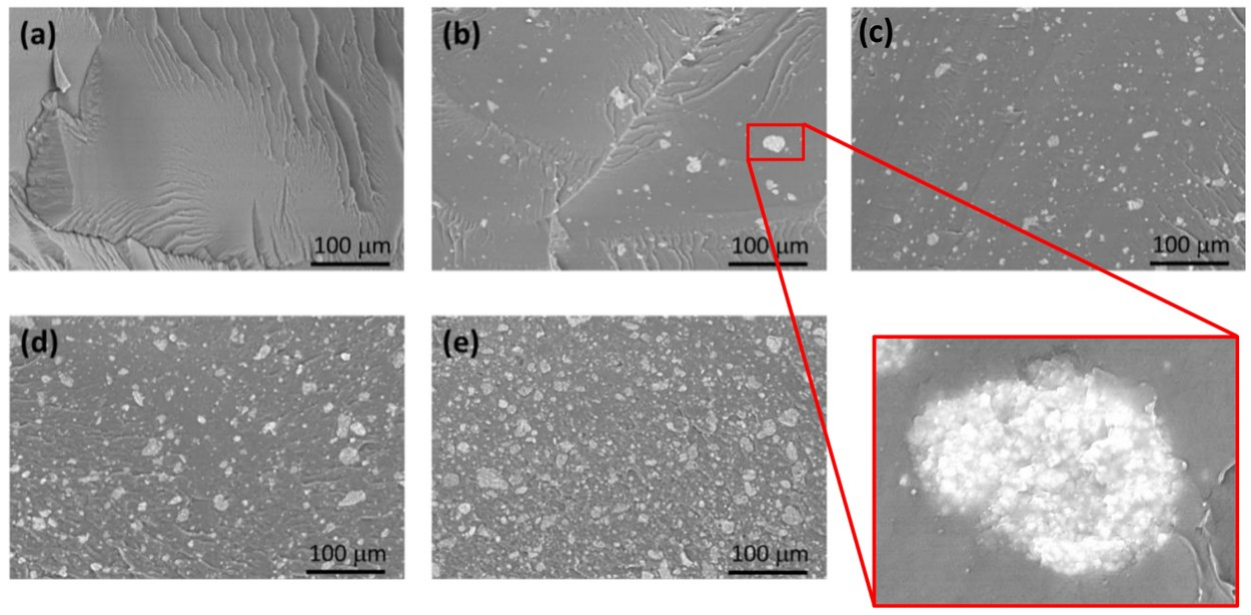
SEM images of composites with different filler content: a) pristine PBAT, b) PBAT+ZTC 5%, c) PBAT+ZTC 10%, d) PBAT+ZTC 20%, and e) PBAT+ZTC 40%. The inset reports the details of the filler–matrix interface.

In Figure 3, the SEM images of the composite microstructure at different ZTC content are reported. The acquisitions are related to the specimen cross-section, obtained by brittle fracture under cryogenic conditions. Since the images are taken using backscattered electrons, the dark gray background corresponds to the polymer matrix, while the lighter particles refer to the ZTC particles, which show good dispersion and distribution within the polymer matrix. In particular, the inset of Figure 3(b) reveals good adhesion at the interface between PBAT and the ZTC particle surface, demonstrating the effective reinforcement by the filler particles due to the presence of filler–matrix interactions and confirming the hypothesis already expressed to explain the viscoelastic behavior of the composites.

**Figure 4:**
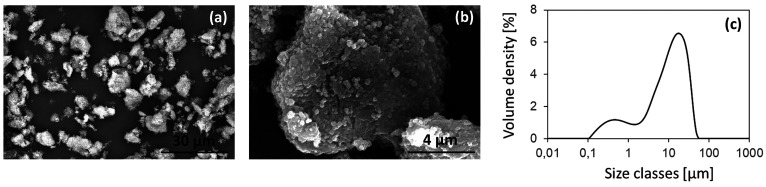
SEM images of ZTC powder at two different magnifications, (a) and (b), and relative particle size distribution (c).

The ZTC particles, after grinding and sieving, show an irregular geometry and an average size less than 30 μm (Figure 4(a)), confirmed by the particle size distribution with a *D10*, *D50*, *D90*, and *D*_*mean*_ equal to 0.7 ± 0.1, 10.7 ± 0.5, 29 ± 3, and 13 ± 1, respectively (Figure 4(c)). Each ZTC grain also appears to consist of an aggregate of smaller particles, presumably TiO_2_, held together by the zein protein structure. The surface of each particle thus appears extremely jagged and wrinkled (Figure 4(b)).

**Figure 5:**
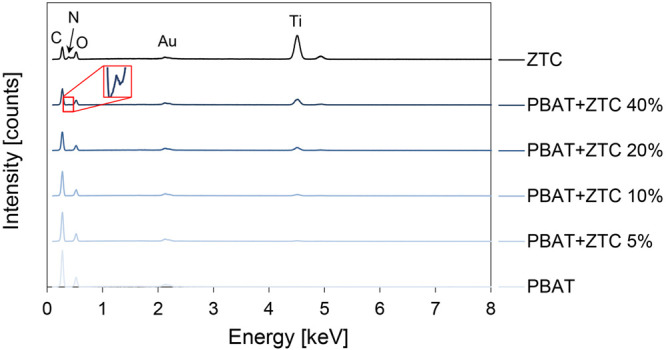
EDS spectra of PBAT-based composite materials and the ZTC complex.

The material composition is confirmed by the elemental analysis reported in Figure 5. From the spectra comparison, the increase in ZTC content affects the intensity of the characteristic peaks associated with pure PBAT. In particular, the decrease in the peak associated with carbon (C) and the increase in the titanium (Ti) peak are observed as the filler percentage in the composite increases. The nitrogen (N) peak is also discernible in the PBAT+ZTC 40% sample.

The intensity of the oxygen (O) peak, on the contrary, does not substantially change as the polymer and ZTC contribution, which respectively increases and decreases, is equivalent.

### 3.3 IR Spectroscopy Characterization

**Figure 6:**
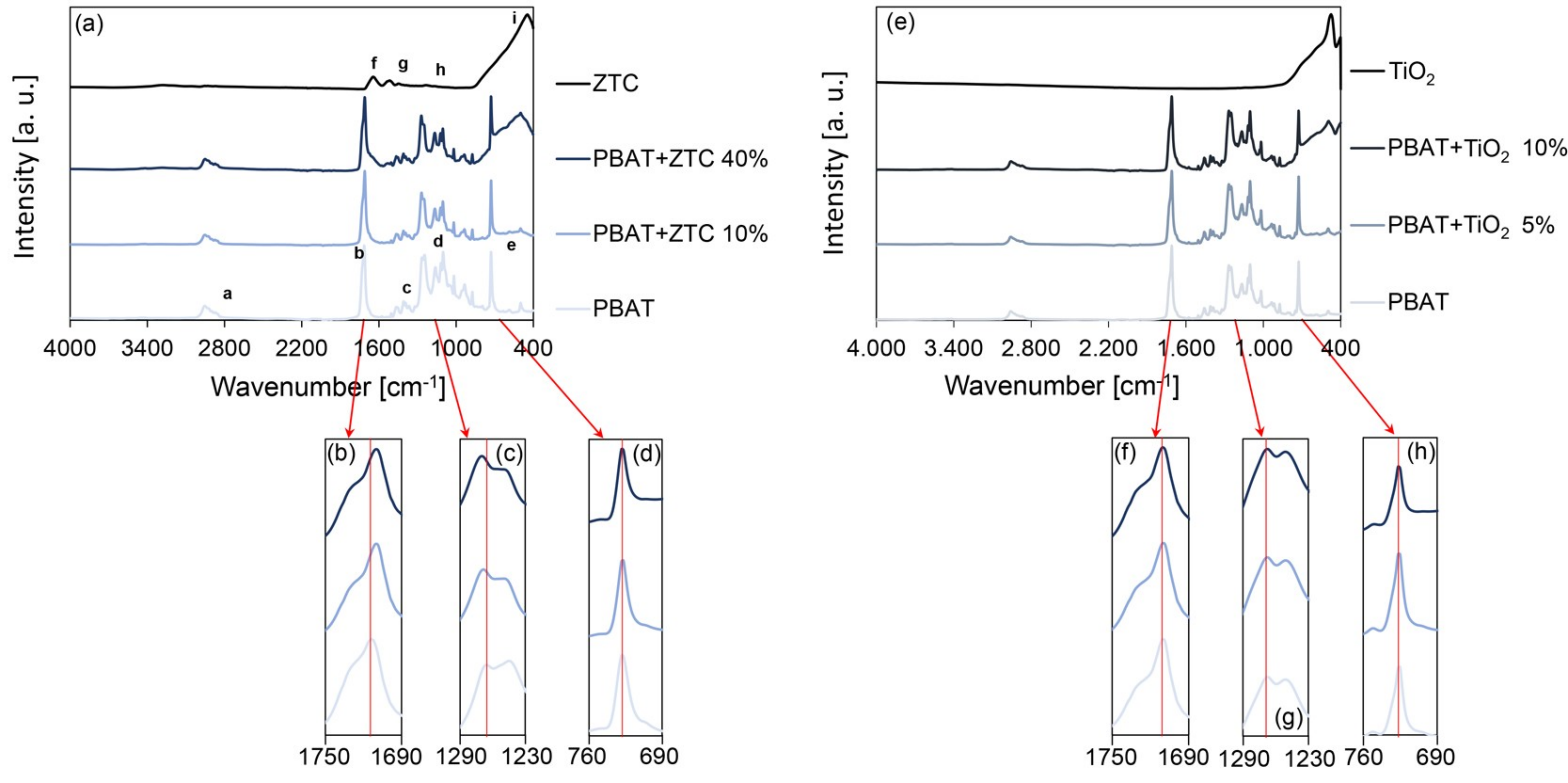
IR spectra of pristine PBAT, PBAT+ZTC 10%, and PBAT+ZTC 40% composites as representative samples and ZTC powder (a). In the insets (b), (c), and (d), the exact position of some characteristic peaks for pristine PBAT and PBAT-ZTC composites is highlighted. IR spectra of pristine PBAT, PBAT+TiO_2_ 5%, and PBAT+TiO_2_ 10% composites as representative samples and TiO_2_ powder (e). In the insets (f), (g), and (h), the exact position of some characteristic peaks for pristine PBAT and PBAT-TiO_2_ composites is highlighted.

Figure 6(a) shows the IR spectra of composite samples with increasing ZTC content (pristine PBAT, PBAT+ZTC 10%, and PBAT+ZTC 40% as representative samples) and pure ZTC powder, to detect any structural changes in the macromolecules due to the interaction with the ZTC particles.

In the ZTC spectrum, the bands at 1644, 1516, 1233, and 446 cm^–1^ can be identified (Figure 6(a)). For the zein structure, the 1644 (amide I) cm^–1^ band can be assigned to C=O stretching, while the bands at 1516 (amide II) and 1233 (amide III) cm^–1^ cannot be assigned to motions of individual bonds as they both involve C–N stretching and N–H in-plane deformation.^34,35^ These absorption bands are common to all proteins and polypeptides^36^ and are related to some normal modes of the peptide group −C(=O)–N(−H)–. The broad band, spiked at 446 cm^–1^, is assigned to Ti–O–Ti stretching in the TiO_2_ network.^37,38^

The characteristic peaks of pristine PBAT, PBAT+ZTC 10%, and PBAT+ZTC 40% composites, as representative samples (Figure 6(a)), are summarized in Table 2. As the filler concentration increases, the appearance of the ZTC distinctive peaks in the PBAT spectrum can be noted. It can also be observed that the ZTC presence in the polymer matrix results in a red shift (from 1714 to 1709 cm^–1^) for the absorption frequency of the PBAT carbonyl (C=O) group, because of the hydrogen bond formation between the oxygen atom in the polymer C=O groups and the amine (−NH–/–NH_2_) or hydroxyl (−OH) hydrogen in the zein structure^39^ (Figure 6(b)). The hydrogen bond development between zein and other polymeric structures has already been observed in systems containing macromolecular structures, such as cellulose^35^ and starch.^40^ The presence of these intermolecular bonds represents the driving force to generate good adhesion at the interface between PBAT and the ZTC particle surface. When a hydrogen bond is formed, the carbon atom in the C=O group is depleted of electrons and partially positively charged, resulting in an increase of its electronegativity.

**Table 2:** Characteristic Wavenumbers of IR Peaks for PBAT and ZTC

parameter	band	remarks
a	2953–2874 cm^–1^	–CH_2_– symmetric and asymmetric stretching in the BA unit
b	1714 cm^–1^	–C=O stretching in the BT and BA units
c	1578–1453 cm^–1^	–C=C– stretching in the terephthalate aromatic ring
d	1265–1246 cm^–1^	(O=)C–O– symmetric and asymmetric stretching in the BT and BA units
e	728 cm^–1^	out-of-plane =C–H bending in the terephthalate aromatic ring
f	1644 cm^–1^	amide I of zein protein
g	1516 cm^–1^	amide II of zein protein
h	1233 cm^–1^	amide III of zein protein
i	446 cm^–1^	Ti–O–Ti stretching in the TiO_2_ network

This condition would affect the covalent bond formed by the other oxygen in the ester group (C–O), with the bond strength increase. The consequence is a blue shift of the C–O stretching from 1265 to 1270 cm^–1^ (Figure 6(c)). The meaningfulness of these shifts, in which the ester group is involved, is confirmed by the fact that the peak related to the aromatic out-of-plane C–H bending of the terephthalic unit (728 cm^–1^), not implied in hydrogen bonding, is not displaced (Figure 6(d)). The hydrogen bond development confirms the effectiveness of the interactions at the PBAT-ZTC interface, endorsing the results already highlighted by the improved mechanical properties and the microstructure homogeneity of the composites.

To verify that the formation of hydrogen bonds is due to the compatibilization effect of the zein, for comparison, equivalent systems containing only PBAT and TiO_2_ (in the same quantity as in ZTC) were prepared and characterized by IR spectroscopy (Figure 6(e)). In this case, the C=O stretching peak, characteristic of PBAT, does not red shift (Figure 6(f)) and remains in the same position even in the presence of TiO_2_. The same applies for the C–O stretching peak in the ester bond, which does not show blue shift (Figure 6(g)), while the peak of the C–H aromatic bending remains constant (Figure 6(h)), thus validating the previous observations. Not showing the presence of interactions at the interface, systems with PBAT and TiO_2_ were not further investigated in this work.

### 3.4 FDM 3D Printing

**Figure 7:**
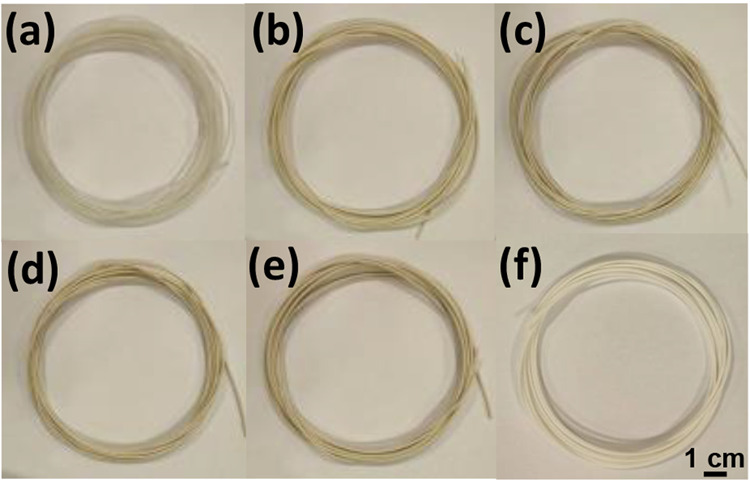
FDM filaments of PBAT (a), PBAT+ZTC 5% (b), PBAT+ZTC 10% (c), PBAT+ZTC 20% (d), and PBAT+ZTC 40% (e) and commercial PLA (f) as a comparison.

To demonstrate the printability of PBAT-based composites in 3D objects, the biocomposite filaments with different ZTC content were first extruded (Figure 7) and then used as starting materials for FDM printing of solid structures. Using the optimized extrusion conditions, no variations in extrusion flow were observed, and a total length of about 30 m of homogeneous filaments with constant diameter was obtained for each composition. Due to the high ZTC fraction used with the PBAT matrix, the filament obtained from the PBAT+ZTC 40% formulation appeared rougher on the surface.

**Figure 8:**
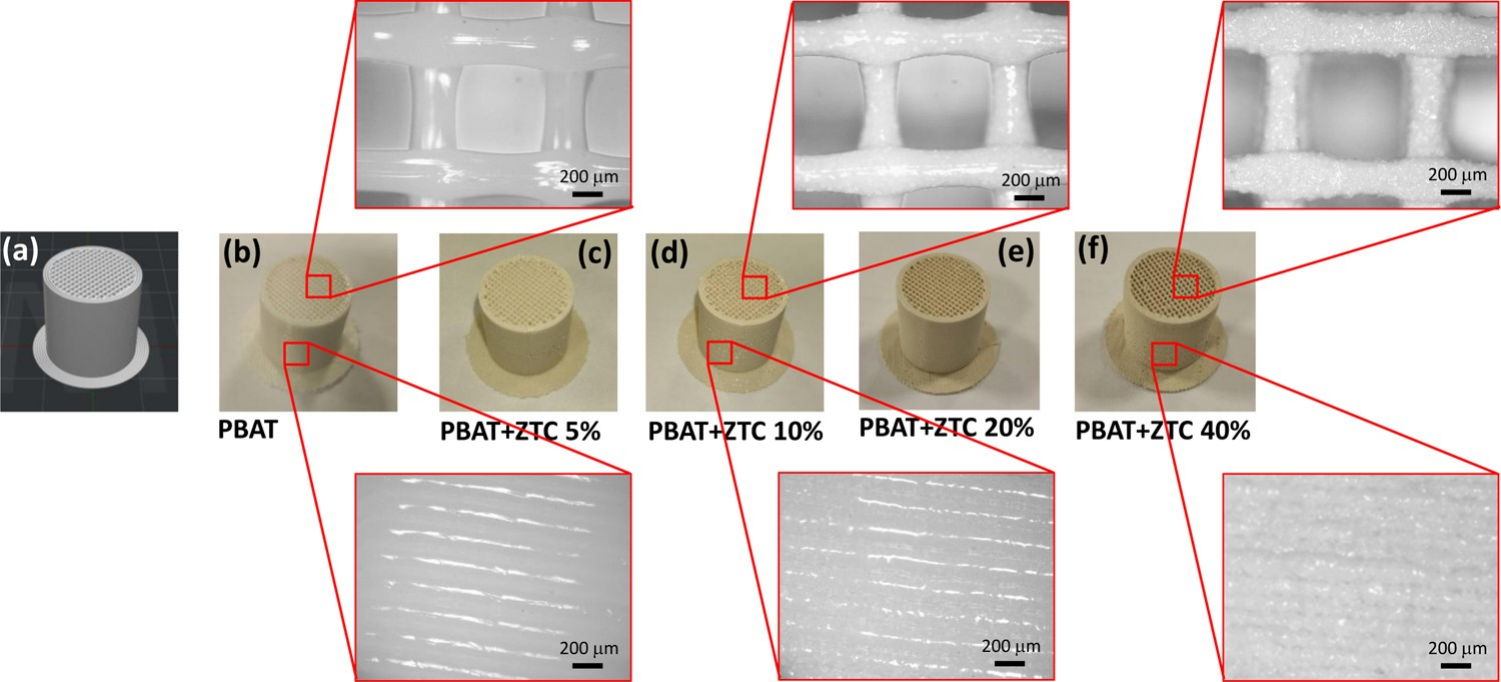
FDM 3D printed scaffolds: 3D model (a), pristine PBAT (b), PBAT+ZTC 5% (c), PBAT+ZTC 10% (d), PBAT+ZTC 20% (e), and PBAT+ZTC 40% (f). In the insets, the details of the infill pattern and the lateral layering for some representative samples.

Cylindrical scaffolds, with the filling pattern obtained by alternating the direction of filament deposition between 0 and 90°, were printed for each composite formulation (Figure 8(b–f)). The 3D model is also shown in Figure 8(a). The printed scaffolds correspond to the designed model from the dimensional and geometric point of view, with similar performances in terms of the filament consistency for the pure PBAT and the PBAT-based composites at different ZTC content.

The optical microscope (Optika B-380 series microscope, Optika Srl, Italy) images allowed observing with more detail the scaffold internal structure and the regularity of the deposited strand, indicating the good dimensional stability of the pores and continuity of the strand generated by the filament during extrusion.

The strand shape shows a circular cross-section of approximately 400 μm, without collapsing in the unsupported segment (upper inset of Figure 8). The scaffold side wall provides information regarding the layer stratification (lower inset of Figure 8): the adjacent strands are in mutual contact, reaching a good degree of interlayer coalescence, and no delamination is observed.^41^

**Figure 9:**
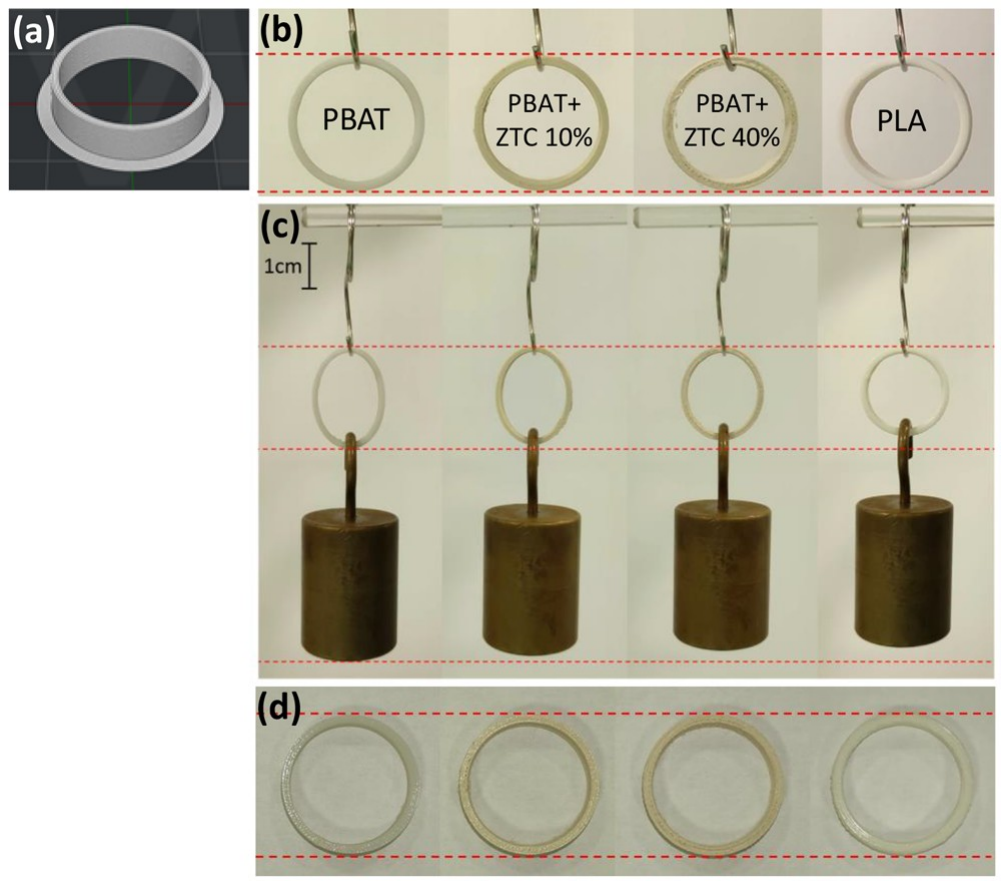
FDM 3D printed rings: 3D model (a); pristine PBAT, PBAT+ZTC 10%, and PBAT+ZTC 40% as representative samples and PLA as reference (b). Ring elastic deformation due to the force application (c) and deformation recovery after force removal (d).

To demonstrate the possibility of tailoring the flexibility through the filler content in the polymer matrix, ring structures were printed (Figure 9(b)) and subsequently subjected to elastic deformation by applying a mass of 200 g, corresponding to a weight force of 1.962 N (Figure 9(c)). The ring 3D model is also shown in Figure 9(a).

The ring deformation gradually decreases as the filler content increases, giving the chance to tailor the properties of the printed object according to the application needs. As expected, the PLA ring did not show any noticeable deformation. Finally, the deformation was completely recovered once the load was removed (Figure 9(d)).

**Figure 10:**
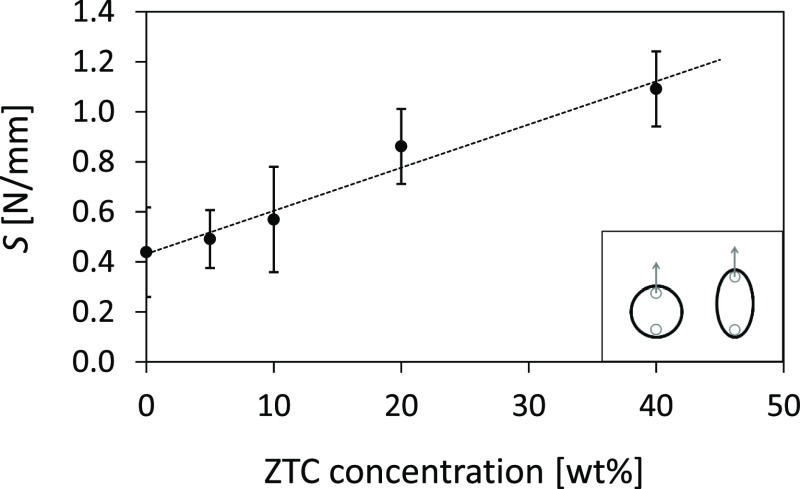
*S* values as a function of the ZTC content. In the inset, the schematization of the analyzed ring deformation.

To quantify the load required to elliptically stretch the ring, the 3D printed structures were subjected to tensile tests using a specific configuration of the dynamometer. The rings were placed around custom-made steel bars connected to the load cell and deformed at a constant speed (50 mm/min) starting from a condition of circularity without stress on the ring, as outlined in the inset of Figure 10. The stiffness (*S*) [N/mm] of the rings was calculated from the slope of the linear starting segment of the load–displacement curve and represents the load necessary to geometrically deform along the tensile axis the ring, which assumes an increasing ellipticity as the test proceeds.^42^ The *S* value increases with increasing the ZTC content, as shown in Figure 10, ranging from 0.44 ± 0.18 to 1.09 ± 0.15 [N/mm] for the pure PBAT and PBAT+ZTC 40% ring, respectively, thus confirming the qualitative data shown in Figure 9.

### 3.5 Biocompatibility Test

**Figure 11:**
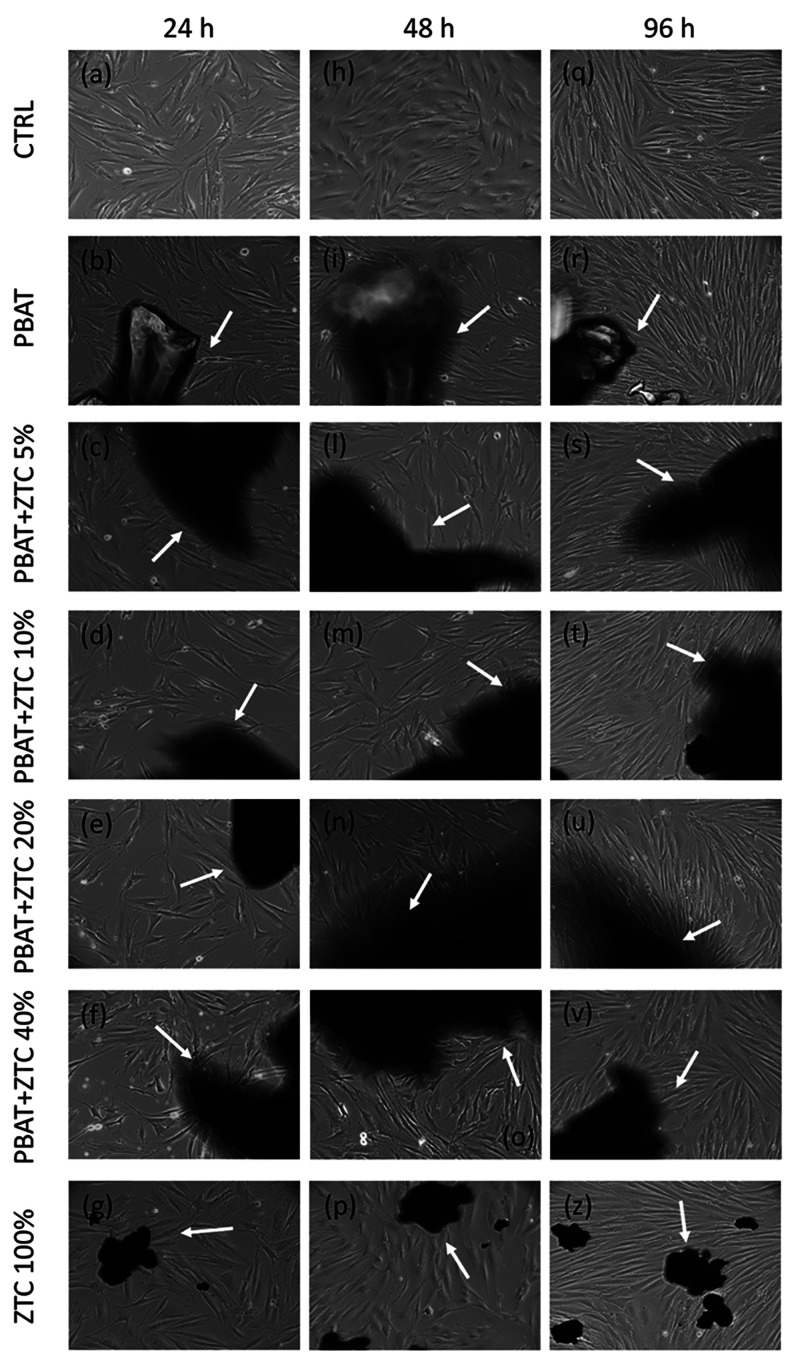
TLM images of HDFs grown for 24 (a–g), 48 (h–p), and 96 h (q–z). Untreated HDFs (CTRL), pure PBAT, PBAT+ZTC from 5 to 40%, and ZTC powder 100% are shown. Optical magnification is 10×. White arrows indicate normal shaped, healthy HDFs that grow in close proximity of PBAT, PBAT-based composites, and ZTC.

To evaluate the biocompatibility of PBAT-based composites, 2.5 g/mL biocomposite small grains with different ZTC content were added to cultured HDFs, and the effects on cell morphology, growth, and viability were evaluated. In Figure 11(a–z), transmitted light microscopy images of HDFs grown in the absence (CTRL) or presence of ZTC powder, PBAT, and PBAT-composites at different ZTC content are reported. In the images, the dark areas precisely indicate the material grains put in contact with the cell culture. HDFs grown up to 96 h in direct contact with the tested materials show a morphology identical to that of CTRL cells and do not show any sign of cell stress. The images reported in Figure 11 do not objectively show significant changes in the cell proliferation of HDFs grown in direct contact with PBAT-composites compared to the CTRL, irrespective of cell culture time or ZTC content. Actually, HDFs divide normally as indicated by the increased confluence of cells grown for 48 and 96 h compared to 24 h, which do not differ from CTRL.

To evaluate more thoroughly the cytotoxicity of PBAT-composites, the effects of these materials on cell viability were measured by a high sensitive luminescence-based cell viability assay that quantifies the intracellular ATP content. ATP is a biomolecule directly related to the metabolic activity of the cells; therefore, the luminescence readout of the in vitro test is directly proportional to the number of viable cultured cells.

**Figure 12:**
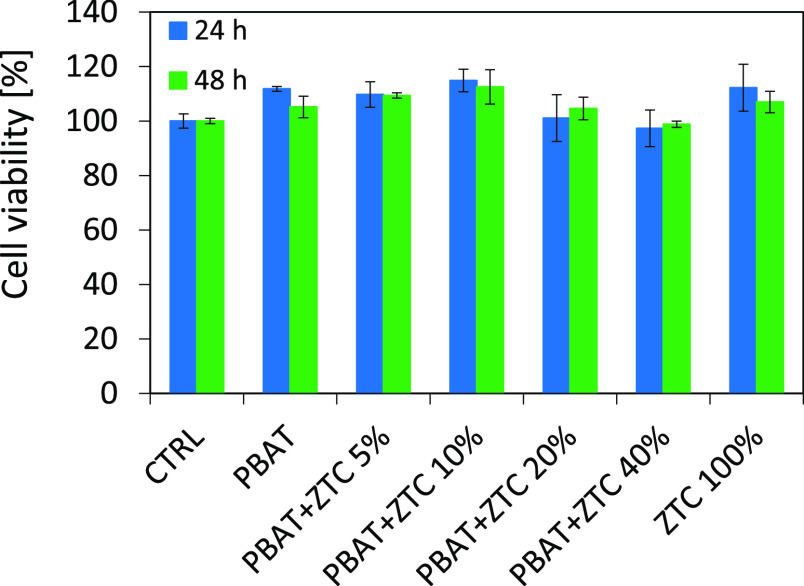
Cell viability of HDFs grown for 24 h (blue bars) or 48 h (green bars) in the presence of pure PBAT, PBAT-based composites (from 5 to 40%), and ZTC 100%. Values are expressed as % of the viability measured in the HDFs control sample (CTRL) that has a 100% cell viability reference value.

The experimental mean values of the HDFs viability among the various samples analyzed are reported in Figure 12, and they are not to be considered statistically different both at 24 and 48 h, as the ANOVA test results show a P-value of 0.034 and 0.028, respectively, for the viability at 24 and 48 h. Both P-values are higher than the level of significance chosen (α = 0.01), and according to the obtained results, the composite materials can be considered noncytotoxic as they do not alter the HDFs viability compared to the control.^43^

The flexible behavior and biocompatibility of these biobased formulations open new application fields, such as biomedicine,^44^ pharmaceutics,^45^ sensing,^46^ and robotics,^47^ for FDM 3D printing technology, which is currently constrained by low sustainable and eco-friendly materials for obtaining soft systems.^25^

Advanced solutions such as anatomical models and medical training systems,^48^ drug delivery systems,^49^ customized laboratory devices,^50^ or tissue engineering scaffolds,^51^ inserted directly into a biologically active environment, can be successfully realized using the FDM 3D approach with the selection of the appropriate material. Particularly interesting is the possibility of printing organs for improving the realism in surgical practice, where there is the need to have materials with different stretching and bending properties depending on the consistency of the tissues to be reproduced.^52^

## 4 Conclusion

A successful method for preparing FDM 3D printed PBAT-based biocomposite filaments is described. Different formulations, with reinforcing content up to 40 wt % of the ZTC complex, were obtained via the solvent casting approach. The procedure allowed for effective enhancement of viscoelastic and thermo-mechanical properties of pristine PBAT. The ZTC complex, based on zein and titanium dioxide, produced in the polymeric matrix an improvement of the storage modulus (*E*′ increased from 200 to 590 MPa at 20 °C). In addition, the compliance *J*(*t*) decreased from 8.1 down to 2.3 GPa^–1^ at 20 °C, reducing the polymer chain mobility and stepping up the interaction density at the filler–matrix interface. This condition allows for an extension of the application range for these materials from a thermal and temporal point of view.

SEM images of sample microstructures showed that the filler particles were homogeneously dispersed and distributed within the polymer network at each ZTC concentration, without phase segregation at the micrometric level. No separations were evident at the ZTC-PBAT interface even at high magnifications.

IR spectra revealed the formation of hydrogen bonds between the polymer chains and presumably the protein structures of the ZTC complex, underlining the coupling effect assumed by the zein in increasing the affinity between the PBAT matrix and inorganic filler in the developed composite system. The spectroscopy results confirmed the thermo-mechanical and structural characterizations.

From these composite materials, constant size filaments were experimentally obtained and used for FDM 3D printing of different solid structures.

Preliminary cytotoxicity assay did not show any detrimental effects of PBAT-based composites after direct contact with in vitro cultured HDFs, according to transmitted light microscopy observations and cell viability assay.

Obtained data support the idea that PBAT-based composites with different ZTC content combine the tunable mechanical properties, sustainable eco-designs, and the potential of additive manufacturing properties with short time, direct-contact biocompatibility, paving the way toward wide possibilities of advanced biomedical applications.
